# Oral microbiota and *Helicobacter pylori* in gastric carcinogenesis: what do we know and where next?

**DOI:** 10.1186/s12866-021-02130-4

**Published:** 2021-03-04

**Authors:** Seyedeh Zahra Bakhti, Saeid Latifi-Navid

**Affiliations:** Department of Biology, Faculty of Sciences, University of Mohaghegh Ardabili, Ardabil, 56199-11367 Iran

**Keywords:** Oral microbiota, *H. pylori*, CagA, Interaction, Gastric cancer

## Abstract

Gastric cancer (GC) is one of the most common malignancies causing death worldwide, and *Helicobacter pylori* is a powerful inducer of precancerous lesions and GC. The oral microbiota is a complex ecosystem and is responsible for maintaining homeostasis, modulating the immune system, and resisting pathogens. It has been proposed that the gastric microbiota of oral origin is involved in the development and progression of GC. Nevertheless, the causal relationship between oral microbiota and GC and the role of *H. pylori* in this relationship is still controversial. This study was set to review the investigations done on oral microbiota and analyze various lines of evidence regarding the role of oral microbiota in GC, to date. Also, we discussed the interaction and relationship between *H. pylori* and oral microbiota in GC and the current understanding with regard to the underlying mechanisms of oral microbiota in carcinogenesis. More importantly, detecting the patterns of interaction between the oral cavity microbiota and *H. pylori* may render new clues for the diagnosis or screening of cancer. Integration of oral microbiota and *H. pylori* might manifest a potential method for the assessment of GC risk. Hence it needs to be specified the patterns of bacterial transmission from the oral cavity to the stomach and their interaction. Further evidence on the mechanisms underlying the oral microbiota communities and how they trigger GC may contribute to the identification of new prevention methods for GC. We may then modulate the oral microbiota by intervening with oral-gastric bacterial transmission or controlling certain bacteria in the oral cavity.

## Background

The oral microbiota is a complicated ecosystem in the body. More than 700 bacterial species live in the human oral cavity, which include 11 bacterial phyla and 70 genera [1]. The main phyla of oral bacteria include *Fusobacteria, Firmicutes*, *Proteobacteria*, *Bacteroidetes*, and *Actinobacteria* [2]. The composition of the oral microorganisms can be associated with the carcinogenesis of distant organs, especially the gastrointestinal (GI) tract. Many studies have provided evidence that oral microbiota play major roles in GI cancers [3–5]. Species, such as *Tannerella forsythia, Porphyromonas gingivalis*, *Prevotella intermedia*, *Parvimonas,* and *Leptotrichia* were correlated with the risk of various kinds of GI cancers [6–10]. Gastric cancer (GC) is one of the most common malignancies causing death. The direct relationship between oral microbes and the GC risk has not been completely assessed [11]. Microbial communities are considered an important factor in the incidence and development of GC [12]. The GC microbiome has been characterized by the enrichment of numerous bacterial genera and species, which often colonize the oral cavity as opportunistic pathogens or commensals [13]. *Streptococcus, Lactobacillus* [14–17], and *Lactococcus* [15] species were more common in patients with GC [11]. Relative abundance of *Streptococcaceae* family was greater in patients with GC than in other patients [17–19]. *Helicobacter pylori* is a powerful inducer of precancerous lesions and GC [20–25]. Shifts in nutrient availability and gastric acidity and the innate immune response disrupt microbial ecological balance in GC patients, contributing to the overgrowth and colonization of non-*H. pylori* bacteria [26]. This study was set to review the investigations done on oral microbiota and analyze various lines of evidence regarding the role of oral microbiota in GC, to date. In this regard, the possible roles of oral microbiota in GC, the effects of oral microbiota on metabolic pathways and carcinogenic induction, the interaction and relationship between *H. pylori* and oral microbiota in GC, as well as the current understanding with regard to the underlying mechanisms of oral microbiota in carcinogenesis are discussed.

## Main text

### The relationship between oral microbiota and GC

Research studies have proved that oral pathogens are necessary in the GC development (Table 1). It has been shown that changes in the volume of oral microbiota may affect maintaining the local microenvironment that is linked with the progression or development of GC [12]. Applying 16S rRNA marker gene analysis, studies have indicated a significant enrichment of oral-related bacteria in GC [15, 19, 31]. It has been found that the microbial composition of GC patients was significantly different from that of control group [13]. The oral cavity bacterial species including *Leptotrichia*, *Fusobacterium*, *Haemophilus*, *Veillonella*, and *Campylobacter* have higher relative abundances in patients with GC from Singapore and Malaysia compared to others [15]. The most taxa abundant in GC are related to the opportunistic pathogens or commensals that often colonize the oral cavity, such as genera *Aggregatibacter, Alloprevotella*, and *Neisseria*; species *Streptococcus mitis/oralis/pneumoniae*; and also strain *Porphyromonas endodontalis.t_GCF_*000174815 [13]. At the phylum level, the relative frequency of *Firmicutes* was significantly higher while the relative frequency of *Bacteroidetes* was lower in the patients with GC compared to healthy individuals (*P*_*adj*_ for BH = 0.005 and 3.6e-5, respectively). In genus level, *Streptococcus* and *Abiotrophia* had higher relative abundances in GC patients increasing its risk (*P* = 0.0045 and 0.0045 for BH correction, respectively). While genera such as *Prevotella*7, *Neisseria*, *Prevotella*, *Porphyromonas,* and *Haemophilus* reduced the risk of stomach cancer (*P* = 1.89e-04, 9.33e-04, 3.24e-05, 0.002, and 0.022, respectively) [11]. A considerable rise in the relative excess of lactic acid (*Lactobacillus* and *Lactococcus* [15]) was detected in GC patients. Furthermore, it was revealed that *Lactococcus* OTU0002 has powerful cooccurrence interactions with other OTUs related to GC (*Bacillus* OTU0046 and *Aneurinibacillus* OTU0038). Previous studies have similarly reported an increase in *Lactobacillus* species abundance in GC [14, 16, 17]. Bacterial taxa including *Streptococcus anginosus_*OTU68 (*q* = 0.033), *Peptostreptococcus_*OTU16 (*q* = 0.03)*, Gemella_*OTU17 (*q* = 0.033), *Fusobacterium_* OTU33 (*q* = 0.04), and *Slackia_*OTU174 (*q* = 0.033) were enriched in GC [19].

**Table 1:** Direct relationships of oral microbiota with gastric cancer

Oral microbiota (genera/ species)	Country	ASR^**a**^-Both sexes (GLOBOCAN 2012)	Study (Reference)
***Fusobacterium*,** ***Veillonella*,** ***Leptotrichia*,** ***Haemophilus*,** ***Campylobacter, and Lactococcus***	Singapore and Malaysia	8.2 and 7.8	Castaño-Rodríguez et al., 2017 [15]
***Lactobacillus***	South Korea	41.8	Eun et al., 2014 [17]
***Lactobacillus coleohominis*** **and** ***Lachnospiraceae***	Mexico City	6.9	Aviles-Jimenez et al., 2014 [14]
***Lactobacillus***	China	22.7	Wang et al., 2016 [16]
***Clostridium*** **and** ***Fusobacterium***	Taiwan		Hsieh et al., 2018 [27]
**genera** ***Neisseria*,** ***Alloprevotella*, and** ***Aggregatibacter*, species** ***Streptococcus_mitis_oralis_pneumoniae***	China	22.7	Hu et al., 2018 [13]
***Prevotella*** **and** ***Aggregatibacter***	China	22.7	Sun et al., 2018 [28]
***Streptococcus anginosus_*OTU68,** ***Peptostreptococcus_*OTU16 (*P. stomatis*)*, Gemella_*OTU17,** ***Fusobacterium_*** **OTU33, and** ***Slackia_*OTU174 (*S. exigua*)**	China	22.7	Coker et al., 2018 [19]
***Streptococcus*** **and** ***Abiotrophia***	China	22.7	Wu et al., 2018 [11]
***Streptococcus*** **(*Streptococcus mitis*) and** ***Neisseria*** **(*Neisseria flavescens and Neisseria perflava*)**	China	22.7	Liu et al., 2018 [29]
***Lactobacillus*** **sp.,** ***Clostridium*** **sp., and** ***Phyllobacterium*** **sp.**	Portugal	13. 1	Ferreira et al., 2018 [30]

^a^Age-standardized (World) incidence rates

It has been shown that the composition of gastric microbiota varies among the residents of the two cities of Colombia (high-risk Túquerres and low-risk Tumaco). A *Veillonella* sp. and *Leptotrichia wadei* (OTUs: operational taxonomic units) in Túquerres, and *Staphylococcus* sp. in Tumaco were significantly more abundant [32]. In one study, the LEfSe analysis on OTUs revealed that high abundant OTUs such as *Serratia marcescens, Flavobacterium*, *Stenotrophomnonas*, *Klebsiella*, *Pseudomonas*, and *Achromobacter* were enriched in GC samples compared with other samples [33]. Although recent studies have examined the relationship between *Lactobacillus, Fusobacterium, Peptostreptococcus*, and *Streptococcus* in GC patients compared with the control group [15, 27], there is little information about the composition of the microbiota structure with oral origin in GC tissue samples compared to adjacent non-tumor tissues (ANTTs). A study from China showed that the bacterial taxa in the samples of cancer were predominantly represented via oral bacteria (e.g., *Streptococcus*, *Peptostreptococcus*, *Fusobacterium*, and *Prevotella*), but lactic acid-producing bacteria (e.g., *Lactobacillus brevis* and *Lactococcus lactis*) and *Serratia* were more plentiful in ANTTs [12]. The results of LEfSe analysis showed that 33 taxa were enriched in the cancer subjects, like the genera *Prevotella*, *Prevotella_7*, *Peptostreptococcus*, *Streptococcus*, *Selenomonas*, *Acinetobacter*, *Sphingomonas*, *Bacillus*, and *Lachnoanaerobaculum,* and the species *Pseudomonas aeruginosa*, *Acinetobacter baumannii*, *Prevotella oris*, and *Prevotella denticola*; most of them were oral microbiota. Sixteen taxa were also enriched in the non-cancer subjects, like genera *Serratia*, *Lactococcus*, *Helicobacter*, and *Niveispirillum* and the species *L. brevis, S. marcescens*, *H. pylori*, and *L. lactis* [12]. Using the DESeq 2 package, it was shown that the eight genera (*Streptococcus*, *Peptostreptococcus*, *Acinetobacter*, *Sphingomonas*, *Bacteroides*, *Bacillus*, and *Prevotella*_1/_7) were enriched in the cancer subjects. *Fusobacterium* was considerably profuse in cancerous tissues. *Helicobacter* and *Lactobacillus* manifested a significant increase in the ANTTs [12]. Another study showed that, tumor tissue, in comparison to the non-malignant tissues of the stomach, had lower *Proteobacteria* and higher *Bacteriodetes*, *Fusobacteria*, *Firmicutes*, and *Spirochaetes* in Chinese samples. No significant change was observed in phylum-level taxa in Mexican samples [31]. Another study from China showed that merely one bacterial taxa (*Comamonadaceae_*OTU85) overlapped the findings from GC vs. superficial gastritis (SG), depleted in GC lesions in comparison to ANTTs (*q* = 0.024) [19]. Such results highlighted the potential pathogenic impact of the GC-related oral microbiota [12]. Altered GC acidity can increase the chances of oral bacteria colonizing the gastrointestinal tract. Accordingly, the development and occurrence of GC disturb the endogenous bacterial community structure; *H. pylori* may only limitedly affect the progression and/or development of malignant tumors [12].

### Negative link between gastric microbiota with oral origin and GC

Some studies suggest a reversal of oral microbiota in GC (Table 2); for instance, a study from China found that some bacterial taxa including *Acinetobacter_* OTU369 (*q* = 0.045), *Comamonadaceae_*OTU85 (*q* = 0.033), *Candidatus_Portiera_*OTU1596 (*q* = 0.041), and *Vogesella_*OTU661 (*q* = 0.03) were depleted in GC [19]. Bacteria from the *Sphingomonadaceae* family [13, 32], especially *Sphingobium yanoikuyae* species [13], are negatively associated with GC. In the study by Hu et al., analyses at the phyla level showed that the relative abundance of *Proteobacteria* (especially *Neisseria* and *Haemophilus*) in GC subjects was meaningfully decreased in comparison to healthy subjects (*P* < 0.001). In patients with GC compared with healthy controls, it was also shown that the relative frequencies of *Fusobacterium* (*P* = 0.004), *Porphyromonas* (*P* = 0.002)*, Haemophilus* (*P* = 0.007), and *Neisseria* (*P* = 0.008) were significantly reduced [34]. Several studies have shown the significant depletion of genera *Neisseria* in GC [14, 30]. In the study by Avies-Jimenez et al., the species *Streptococcus sinensis* was greatly abundant in NAG compared to MAG-IM and lower in GC [14]. In Korean population, the *L. lactis’s* mean relative abundance was greater in normal control subjects compared to patients with GC [35] (Table 2). Such differences in the relationship between oral microbiota and GC may be due to differences in the populations studied, the kind of samples, the kind of study, the materials and methods used, and the analysis methods.

**Table 2:** Inverse relationship of oral microbiota with gastric cancer

Oral microbiota (genera/ species)	Country	ASR^**a**^-Both sexes (GLOBOCAN 2012)	Study (Reference)
***Neisseria*** **sp.,** ***Streptococcus*** **sp., and** ***Prevotella*** **sp.**	Portugal	13. 1	Ferreira et al., 2018 [30]
***Porphyromonas sp.*,** ***Neisseria sp.,*** **and** ***Streptococcus sinensis***	Mexico City	6.9	Aviles-Jimenez et al., 2014 [14]
***Acinetobacter_*** **OTU369,** ***Comamonadaceae_*OTU85,** ***Candidatus_Portiera_*OTU1596, and** ***Vogesella_*OTU661**	China	22.7	Coker et al., 2018 [19]
***Fusobacterium*,** ***Porphyromonas, Haemophilus*, and** ***Neisseria***	China	22.7	Hu et al., 2015 [34]
***Prevotella7*,** ***Neisseria*,** ***Prevotella*,** ***Porphyromonas,*** **and** ***Haemophilus***	China	22.7	Wu et al., 2018 [11]
***Sphingobium/ Sphingobium yanoikuyae***	China	22.7	Hu et al., 2018 [13]

^a^Age-standardized (World) incidence rates

### Effects of oral microbiota on metabolic pathways and carcinogenic induction

It has been shown that the serological status of bacteria can significantly affect metabolic function. Metabolic contribution of bacteria correlates with carcinogenesis. It has been observed that bacterial metabolic pathways have been significantly increased in GC. The enrichment of carbohydrate absorption and digestion is found to be involved in generating short chain fatty acids (SCFAs) like butyrate, acetate, and propionate plus carbohydrate metabolism pathways in relation with the *Lactococcus* and *Lactobacillus* species enrichment in GC [15]. Castaño-Rodríguez et al., reported several bacterial metabolic pathways that were notably enriched in GC. In addition to carbohydrate metabolism pathways involved in the *Lactococcus* and *Lactobacillus* species enrichment in the GC, they detected the digestion enrichment and carbohydrates’ absorption affecting the SCFAs generation like butyrate, acetate, and propionate. Augmented bacterial SCFA rates may induce colonic cells’ hyperproliferation [36]. A significant rise in the relative lactic acid-producing bacteria’s abundance was seen in GC subjects [15]. Lactate can be a source of energy for the cells of tumor that induce glycolytic enzymes that increase the supply of ATP. This metabolite may potentiate inflammation and activate the angiogenesis of tumor [37–39].

The pathways’ enrichment related with SCFAs’ production in the subjects with GC has been detected by investigating the gastric samples’ microbiome by 16S rRNA marker gene assessment [15, 19]. Many metabolic pathways were significantly enriched in the samples of GC compared with adjacent non-cancerous samples, like those involved in carbohydrate metabolism (e.g., glycolysis and gluconeogenesis), energy metabolism (methane metabolism), and nucleotide metabolism (purine and pyrimidine metabolism) [12]. Purines can regulate immune cell responses and the cytokines release and are rich in the microenvironment of cancer [40]. It has been shown that the purine metabolism pathways are enriched in the cancer subjects [12]. Pathways related to the biosynthesis of L-ornithine, L-arginine, heme, biotin, and lipopolysaccharide (LPS) were enriched in GC group. The enrichment of LPS biosynthesis pathways in GC samples increased microbiota-induced inflammation [13]. LPS has been shown to increase inflammation in the tumor microenvironment and direct tumorigenesis [41, 42]. LPS and *F. nucleatum* cell extracts have been shown to raise inflammatory cytokines and chemokines and create a pro-inflammatory microenvironment that enhances the growth of cancer [43]. Pathways involved in pentose phosphate were predominantly abundant in GC [13]. *S. anginosus*—an oral bacterium—contains the enzyme alcohol dehydrogenase (ADH) that metabolizes alcohol to the carcinogenic acetaldehyde, causing cancer [44]. *S. anginosus* is responsible for inducing the nitric oxide synthesis and inflammatory cytokines causing carcinogenesis [45]. *S. anginosus*—a sulfate-reducing bacterium—affects colonic sulphur metabolism and induces inflammatory cytokines [46]. *P. stomatis*, *P. micra*, *D. pneumosintes*, and *S. exigua* also play a prominent role in progression of GC [19]. The nitrogen-containing compounds’ accumulation like nitrite and nitrate in the stomach may enhance gastric cells’ malignant transformation [47, 48]. *Lactobacillus*, and *Nitrospirae* are described as higher in GC and are involved in nitrate/nitrite metabolism [16]. N-nitroso compounds, which are formed in nitrate/nitrite metabolism, are important carcinogens. Bacteria such as *Haemophilus*, *Staphylococcus, Clostridium*, *Neisseria,* or *Veillonella* may be involved in the formation of these compounds, indicating that they may increase the risk of cancer [48, 49]. Metabolic enzymes associated with denitrification, including nitrous oxide reductase (COG4263) and nitrate reductase (COG1116) were enriched in cancer subjects’ gastric microbiota, compared to the non-cancer group [12].

### Direct relationship between *H. pylori* and oral microbiota in GC

It appears that the *H. pylori* serological status has a notable effect on gastric microbiome α-diversity and composition. The gastric microbiome has been shown to be influenced by *H. pylori* serological status and changed in gastric carcinogenesis [15]. In fact, *H. pylori* affects the structure of the microbial community, and a meaningful increase in alpha diversity has been detected in *H. pylori*-positive samples in comparison with *H. pylori*-negative [12]. Bacterial load was risen considerably in *H. pylori*-positive patients in comparison to *H. pylori*-negative subjects. Infection with *H. pylori* showed a notable effect on bacterial load (*P* < 0.05). Therefore, infection with *H. pylori* might show the bacterial load of the gastric microbiota. This is probably due to variations in the gastric niche caused by *H. pylori*. Shannon’s diversity index in *H. pylori*-positive subjects (2.42 ± 0.58) was increased significantly compared to *H. pylori*-negative subjects (1.56 ± 0.39) (*P* < 0.05) [16]. However, there are studies that show *H. pylori* may be in the oral cavity and has interactions with oral microbes [50–52]. The ability of *H. pylori* to interact with the host and control the local environment was shown with this bacterium’s ability to activate the increased levels of MUC5B and MUC7. Increasing the amount of these oral *H. pylori* receptors may lead to retention and colonization in the oral cavity [53]. *H. pylori* has been observed to have a large capacity to accumulate with *Fusobacterium* spp. isolated from dental plaques (*F. nucleatum* and *F. periodontium*) [52]. In addition, *P. gingivalis* may affect such interactions. Therefore, *H. pylori* is related to the physiological function of *F. nucleatum* and *P. gingivalis* in dental plaque and vice versa [52]. Streptococci—a source of Streptococcus diffuse signal agents (SDSF)—may affect the morphological transformation of *H. pylori* into coccoid forms [54]. *H. pylori* has genes for the absorption and metabolic conversion of D- and L-lactose [55]. In supragingival plaques, the pH buffering process may be mediated in an ammonia-dependent way. *H. pylori* urease converts urea to CO2 and ammonia. Autoinducer-2 (AI-2) is a significant signaling material generated in dental plaque. It is a chemorepellent agent, promoting the *H. pylori* aggregates/biofilms dispersion and initiating negative chemotaxis against the signal source [56]. Therefore, this niche’s *H. pylori* colonization has to be prevented. The factors stimulating coccoid and the low AI-2 levels in supragingival plaque (early- to mid-stages) let dental *H. pylori* establish this niche as nonculturable forms. Subgingival plaque may prefer the mixed spiral and coccoid *H. pylori* populations [52].

It is not yet understood how *H. pylori* affects the structure and diversity of the oral microbiota. However, variations caused by *H. pylori* in the gastric niche affect the growth and colonization of microbes. CagA was associated with increased Gram-negative bacteria in the stomach, hence leading to LPS biosynthesis up-regulation. Through up-regulating LPS biosynthesis in the stomach and attenuating the oral microbiota defense against the microorganisms having a pathogenic potential, infection with *H. pylori* isolates possessing CagA can likely raise the risk of many illnesses [57]. The genera *Actinomyces, Neisseria, Granulicatella, Helicobacter, Veillonella, Streptococcus, Fusobacterium,* and *Prevotella* considerably vary between the *H. pylori-*positive and *H. pylori*-negative sample groups [58]. *Haemophilus, Prevotella, Campylobacter,* and *Veillonella* affect atrophic gastritis activated by *H. pylori* infection [59]. An altered microbial composition with the overgrowth of *Prevotella, Veillonella, Streptococcus*, and *Lactobacillus* was seen in the stomach of *H. pylori*-infected gastric adenocarcinoma and dyspeptic patients [18]. *Neisseria, Haemophilus, Stenotrophomonas*, and *Serratia* dominated the *H. pylori*-negative samples [33]. Significant changes of the gastric microbiota were detected in the *H. pylori* +/ CagA+ samples, and *Helicobacter* and *Haemophilus* genera abundances were increased [57]. The *H. pylori* +/ CagA+ group had greater *Haemophilus* and *Helicobacter* and lower *Roseburia* relative abundances in comparison with other subjects at the genus level [57].

### No or inverse relationship between *H. pylori* and oral microbiome in GC

Infection by *H. pylori* is correlated with the reduced diversity of microbial alpha from *H. pylori*-negative to *H. pylori*-positive with CagA as a notable factor [58]. It has been recently investigated the *H. pylori* impacts on the richness, diversity, and interactions of microbes at the various phases of the disease (i.e. atrophic gastritis, GC, and intestinal metaplasia). Although a decrease in phyllotype richness, diversity, and evenness was reported in *H. pylori*-positive gastric biopsies compared to *H. pylori*-negative samples from chronic gastritis patients, no differences in classification diversity and evenness were seen [59]. This did not change even after controlling for the several stages of GC. However, at all stages the number of interactions between gastric microbes was significantly reduced. Moreover, *H. pylori* presence in superficial gastritis and intestinal metaplasia led to poorer GC-enriched and GC-depleted OTUs interactions, highlighting the potential role of *H. pylori* in alteration of microbial interactions [19].

As stated by Yu et al., oral-associated bacteria composition did not change by *H. pylori* colonization status, however, it changed between tumor gastric and paired non-malignant tissues in Mexican or Chinese samples [31]. *Proteobacteria* (e.g., *Neisseria, Haemophilus, Stenotrophomonas*, and *Serratia*) was the dominant species in the *H. pylori* -negative samples [33]. A study from Japan showed that proportion of *Lactobacillus acidophilus* was greater in *H. pylori* non-infected subjects than individuals with *H. pylori* infection, while the *Lactobacillus salivarius* proportion in *H. pylori*-infected people was high [60]. The relative *Helicobacter* abundance was associated inversely with the *Firmicutes* (*r* = − 0.49; *P* < 0.0001), non-*Helicobacter Proteobacteria* (*r* = − 0.59; *P* < 0.0001), *Actinobacteria* (*r* = − 0.54; *P* < 0.0001), and *Bacteroidetes* (*r* = − 0.43; *P* < 0.0001) abundances [30]. A work from Chile found that among the main phyla of gastric microbiota kept by children, children with *H. pylori* had a relatively lower *Actinobacteria* proportion than non-infected children. The frequency of five genera (i.e. *Actinomyces, Streptococcus, Granulicatella, Rothia,* and an undefined genus in family *Neisseriaceae*) in children with *H. pylori* was significantly reduced compared to non-infected children (*P* = 0.004–0.029). In contrast, the frequency of an unknown genus in the *Comamonadaceae* family was significantly risen in children infected with *H. pylori* versus non-infected children (*P* = 0.014). This reflects the fact that infection with *H. pylori* regenerates gastric microbiota, at least in infection, at several classification levels in children [61].

The relative genus *Streptococcus* abundance was declined markedly in *H. pylori-*positive (*H. pylori*+/CagA− and *H. pylori*+/CagA+) sample groups compared with the *H. pylori*-negative group (*p*_*adj*_ = 0.0216 and 0.0100, respectively) [58]. The relative abundance of *Streptococcus* showed no significant difference between the *H. pylori*+/CagA+ vs. *H. pylori*+/CagA− group (*p*_*adj*_ = 0.1716). Therefore, the expression of *cagA* gene did not affect the colonization of *Streptococcus* gastric [58]. In a study from Colombia, there was no significant association between the total gastric microbiota composition and carriage of the *cag*PAI or *H. pylori* population type. This shows that the changes in gastric microbial composition were highly independent of *H. pylori* colonizing strains. *Streptococcus* and *Neisseria* were genera seen more abundantly in people from the region with low GC risk [32].

### Interaction between *H. pylori* and oral microbiome

Recent evidence suggests that commensal gastric microbes or their metabolites not only affect the ability of *H. pylori* to colonize the stomach but also modulate its pathogenicity potential directly [62, 63]. Many works have shown that infection with *H. pylori* is related with altered gastric microbiota and gastric dysbiosis is involved in some gastric diseases’ pathogenesis. It is not yet known whether *H. pylori* causes the growth of microorganisms or, conversely, the changed microbiota provides good conditions for the colonization of *H. pylori*. It is a two-way interaction; the *H. pylori* colonization prefers the growth of some bacteria, and vice versa, gastric dysbiosis can alter the gastric mucosa or lumen for the colonization of *H. pylori* [62].

It has been shown that *H. pylori* has the potential to alter the interactions between microbes [19]. Zhao et al., revealed that in the oral microbiota of the *H. pylori*-positive group, all interactions were significantly decreased, particularly for people infected with *H. pylori*+/CagA+ strains. Also, the oral microbiota of patients infected with *H. pylori*+/CagA+ was dominated by co-occurrence associations and showed one of the low network complexities because cooperation is destabilizing for the community. Therefore, the oral microbiota of people with *H. pylori*+/CagA+ strains might be more tolerant of alien species’ invasion [57]. *H. pylori* and taxa interactions were co-excluding in the samples of *H. pylori* +/CagA+. Some interactions were common between *H. pylori* − and *H. pylori*+/CagA− sample groups, including co-occurrence between OTU_68_*Roseburia* and OTU_10_*Prevotella copri* and between OTU_68_*Roseburia* and OTU_17_*Propionibacterium*, depleted in the *H. pylori*+/CagA+ group [57]. OTU_30_ *Prevotella_histicola* showed co-occurrence relations with OTU_28*_Prevotella pallens* and OTU_4_*Veillonella dispar,* which were ubiquitous in all subjects. The *H. pylori*+/CagA+ network group was dominated by cooperation associations; only one negative relationship was identified between OTU_11_*Streptococcus* and OTU_3_*Prevotella*. OTU_7_*Roseburia* interactions with OTU_30_ *P. histicola* and OTU_28*_P. pallens* that were detected in the groups representing the *H. pylori* − and *H. pylori*+/CagA−, depleted in the group representing *H. pylori* +/CagA+ [57].

The oral microbiome can possibly affect the bacteria that colonize the stomach. The close relationship between *H. pylori* and *streptococci* was confirmed by the fact that *S. mitis* and *H. pylori* were interacted upon co-cultivation via changed protein biosynthesis in *H. pylori* [64] though not validated under native and acidic conditions. The oral *H. pylori* physiology may potentially have modulated by *Actinomyces* spp. and *Streptococcus* spp.. These microorganisms may inhibit the growth of *H. pylori* in vitro [65]. The compounds secreted by *Streptococcus mutans* [66] and *S. mitis* [67] significantly reduce the durability of *H. pylori*. This effect is due to the *H. pylori* conversion to the nonculturable forms of coccoid. Streptococci SDSF may be involved in *H. pylori* morphological transformation into coccoid forms [54]. Some *Streptococcaceae* strains can have an impact on the final outcomes *H. pylori* infection. In coculture studies *S. mitis* caused the conversion of *H. pylori* to coccoid forms followed by growth inhibition [67]. The *H. pylori* cocooid form (vs. spiral form) shows not only a powerful impact on proliferation but also a poorer impact on apoptosis. The CagA and VacA expressions in the coccoid *H. pylori* were declined in comparison to the spiral form, while VacA was declined greater than that of CagA. The specific inhibitor of ERK1/2 notably blocked the increase in expression in Egr-1 and PCNA induced by the *H. pylori* cocooid form. Thus, the ERK1/2-Egr-1-PCNA pathway activation can affect cell proliferation triggered by cocooid *H. pylori* [68]. Furthermore, this coccoid form’s long latency in gastric mucosa was more associated with the development of GC than the spiral form [68, 69]. It also was shown that many *Lactobacillus* spp. including *Lactobacillus casei, Lactobacillus murinus, L. salivarius* and *L. acidophilis* inhibited *H. pylori* colonization [70–73]. Many *Lactobacillus* spp., as probiotics, can prevent *H. pylori* infection and improve *H. pylori* eradication in humans, although the mechanism is unknown [71]. *L. salivarius* WB 1004 may inhibit the binding of *H. pylori* to the gastric epithelial cells of murine and human and decrease IL-8 release in vitro [74]. *L. salivarius*, but *L. casei* or *L. acidophilus* generates abundant lactic acid as *H. pylori* inhibitor [75]. *Lactobacillus gasseri* OLL 2716 (LG21) has an ability to connect the gastric epithelium and withstand gastric acidity. It suppresses *H. pylori* and reduces gastric inflammation studied by the 13C-urea breath test and the serum pepsinogen levels [76]. Castaño-Rodríguez et al., found that the subject’s *H. pylori* serological status was related to a significant alteration in the predicted global microbial metabolic output. Using LEfSe, it was identified that KEGG (Kyoto Encyclopedia of Genes and Genomes) pathways were enriched across the serological status of *H. pylori*; 20 predicted pathways (KEGG Level 3) were enriched in subjects with GC in comparison to controls. Additionally, carbohydrate absorption and digestion, which are somehow responsible for SCFAs production including propionate, butyrate, and acetate, were also enriched in GC [15].

### Mechanisms underlying carcinogenic activity of oral microbiota

**Fig. 1:**
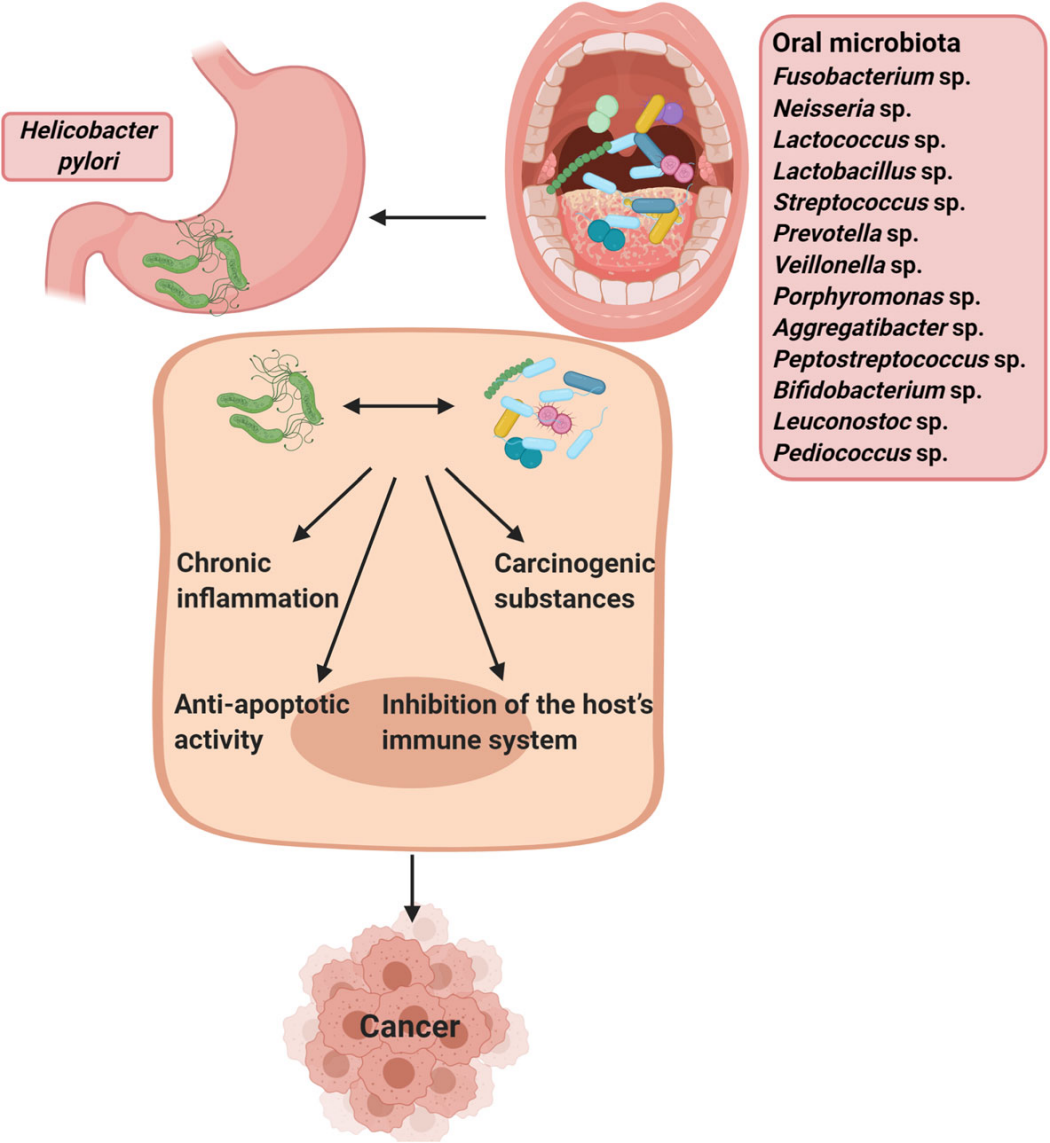
Potential mechanisms of oral microbiota include the induction of chronic inflammation, the inhibition of the host’s immune system, the anti-apoptotic activity, and the carcinogenic substances that may promote cancer

There are numerous potential mechanisms of action of oral microbiota that may cause carcinogenesis: I) Induction of chronic inflammation: Inflammatory mediators produced by oral bacteria, especially *Fusobacterium*, *Porphyromonas*, and *Prevotella,* cause oncogene activation, mutagenesis, DNA damage, cell cycle arrest, cell proliferation, tumor invasiveness, migration, metastasis, and angiogenesis [77, 78]. II) Inhibition of the host’s immune system: Oral microbiota such as *P. gingivalis* [79] and *F. nucleatum* [80–82] protect tumor cells by inhibiting immune responses. III) Anti-apoptotic activity: Oral bacteria such as *F. nucleatum* [83] *and P. gingivalis* [84] causes cancer growth by the activation of anti-apoptotic signaling pathways and inhibiting pro-apoptotic pathways that eventually lead to inhibition of cellular apoptosis. and IV) Carcinogenic substances: Oral bacteria produce some substances that play a role in chronic inflammation, genomic instability, accumulation of mutations, metastasis, and progression of GC [43, 85, 86] (Fig. 1).

#### Chronic inflammation

**Fig. 2:**
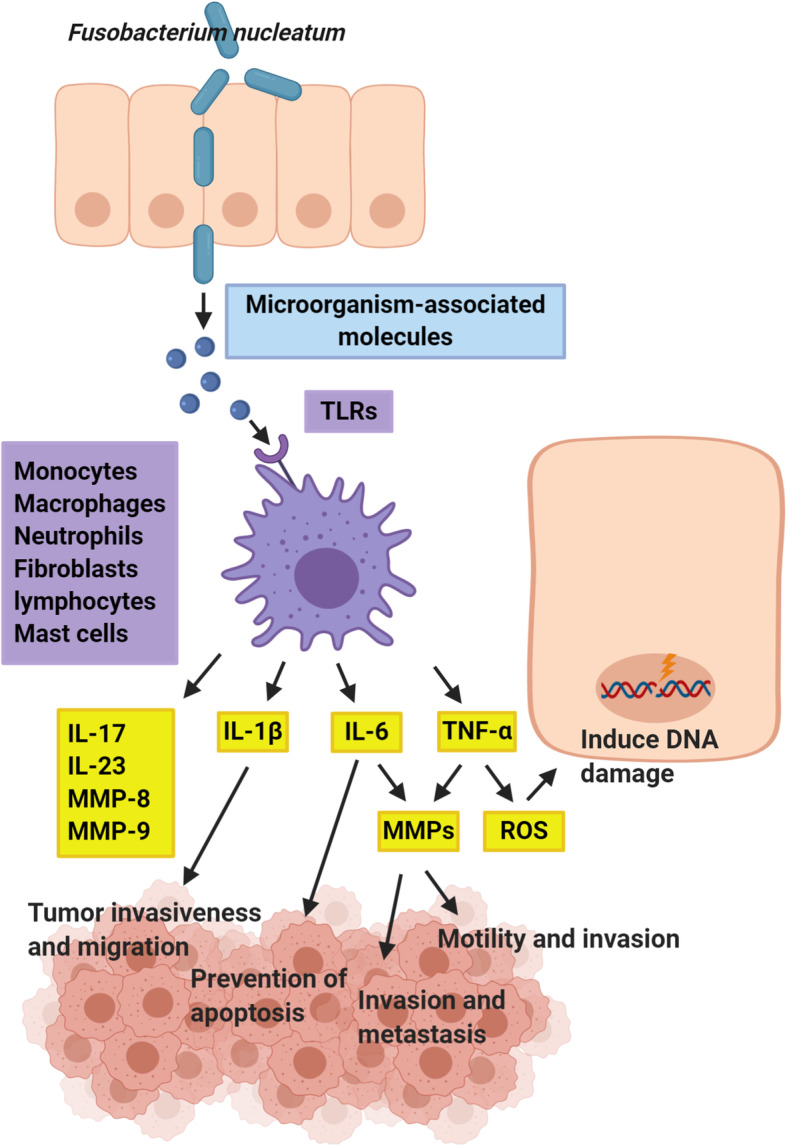
Oral bacteria increase the production of various types of inflammatory mediators such as interleukin-1β (IL-1β), IL-6, IL-17, IL-23, TNF-α, and MMP-8 and MMP-9, which are involved in DNA damage, tumor invasiveness, migration, metastasis and prevention of cell apoptosiss. High content of IL-1β correlates with tumor invasiveness and migration. IL-6 contributes to apoptosis suppression. IL-6 also influences the metastasis and invasion processes via increasing the expression of MMPs. TNF-α may induce DNA damage through generation of ROS. TNF-α can affect invasion and motility processes via inducing expression of MMPs. TNF-α: tumor necrosis factor-α, MMPs: matrix metalloproteinases, ROS: reactive oxygen species

Chronic inflammation is known as the most prominent preventable cause of cancer [85–88]; some inflammatory cytokines may activate oncogenes [85]. Inflammation can also enhance progression of cancer and speed up invasion and metastasis processes [85, 86]. Oral microbiota, especially *Fusobacterium*, *Porphyromonas*, and *Prevotella* (anaerobic species), induce chronic inflammation. These bacteria incite the production of inflammatory mediators and adversely affect epithelial cells and extracellular matrix components. Oral pathogens associated with up-regulation of many cytokines such as interleukin-1β (IL-1β), IL-6, IL-17, IL-23, TNF-α, and other inflammatory mediators such as matrix metalloproteinases (MMPs) MMP-8 and MMP-9 are involved in carcinogenesis [77, 78]. *P. gingivalis* can promote local inflammation contributing to carcinogenesis [79]. Moreover, investigation of the anti-proliferative impact of the *L. lactis* cytoplasmic fraction on cancer cell line indicated a preventive influence on cell multiplication. *L. lactis* induced G0/G1 cell cycle arrest linked to an increase in expressions of p21 and p53, retinoblastoma protein phosphorylation, and a decrease in cyclin D1 expression, hence inducing apoptosis [89]. In recent studies, *Fusobacterium* species have attracted a lot of attention, with autophagy and TLR4 playing a crucial role in the inflammation they cause [90–92]. It has been shown that LPS and *F. nucleatum* cell extracts may raise chemokine and inflammatory cytokines and produce a proinflammatory microenvironment, promoting progression of cancer [43]. *F. nucleatum* can connect to cancerous and natural epithelial cells through FadA connection to E-cadherin [93]. This connection also stimulates β-catenin-regulated transcription, increasing the oncogenes cyclin D1 and c-Myc expression; Wnt signaling genes Wnt7a/Wnt7b/Wnt9a; and inflammatory genes nuclear factor-κB (NF-κB), IL-6, IL-8, and IL-18 which are responsible for carcinogenesis [93, 94]. IL-6 can induce oxidative stress and cause H_2_O_2_ transient accumulation in mitochondria and hence lead to mitochondrial damage [95, 96]. Most genes targeted by IL-6 contribute to the progression of the cell cycle and the suppression of the apoptosis. IL-6 may affect the development of cancer through influencing anti-apoptotic pathways [83]. IL-6 also influences the metastasis and invasion processes via increasing the expression of MMPs [97]. High content of IL-1β correlates with tumor migration, invasiveness, and higher aggressive tumor phenotype [98]. It was associated with lower E-cadherin expression, promoting cell migration [99]. TNF-α is also secreted responding to several factors, such as bacterial LPS. It triggers the generation of reactive oxygen compounds, prostaglandins, metalloproteinases, and leukotrienes [100]. For TNF-α-induced tumor growth, the activation of signaling pathways, such as Wnt and NF-κB, is crucial [101]. Furthermore, TNF-α may induce DNA damage through generation of reactive oxygen species [102]. It can affect invasion and motility processes via inducing expression of MMPs (Fig. 2) [103].

#### Inhibition of the host’s immune system

Progression of cancer might be fueled by host immune system and microbiota interaction, particularly, in the gastrointestinal tract of the human in which there are plenty of bacteria; the immune system is very reactive [42]. Studies have suggested that *P. gingivalis* [79] and *F. nucleatum* [80, 81] could induce inhibition of the host’s immune response, and reducing these bacteria may lead to a decreased inhibition of immune responses. *P. gingivalis* can invade the eukaryotic cells through several virulence mechanisms, such as inhibiting the anti-oxidative and host’s immune systems and increasing the inflammation [104]. *F. nucleatum* has been reported to inhibit the proliferation and induction of T-cell apoptosis by expanding myeloid-derived immune cells [81]. *F. nucleatum* protects tumor cells from immune cell attack and natural killer (NK) -mediated killing [80]. Moreover, *F. nucleatum* can save the cells of the tumor from the immune cell attack and natural killer (NK)-mediated killing by interacting of its Fap2 protein with the inhibitory TIGIT (T cell immunoreceptor with Ig and ITIM domains) on the T and NK cells [80]. Various *F. nucleatum* strains can inhibit the NK cell killing of several tumors. It is mediated by the human TIGIT. The *F. nucleatum* Fap2 protein can directly interact with TIGIT, preventing the NK cell cytotoxicity [82].

#### Anti-apoptotic activity

**Fig. 3:**
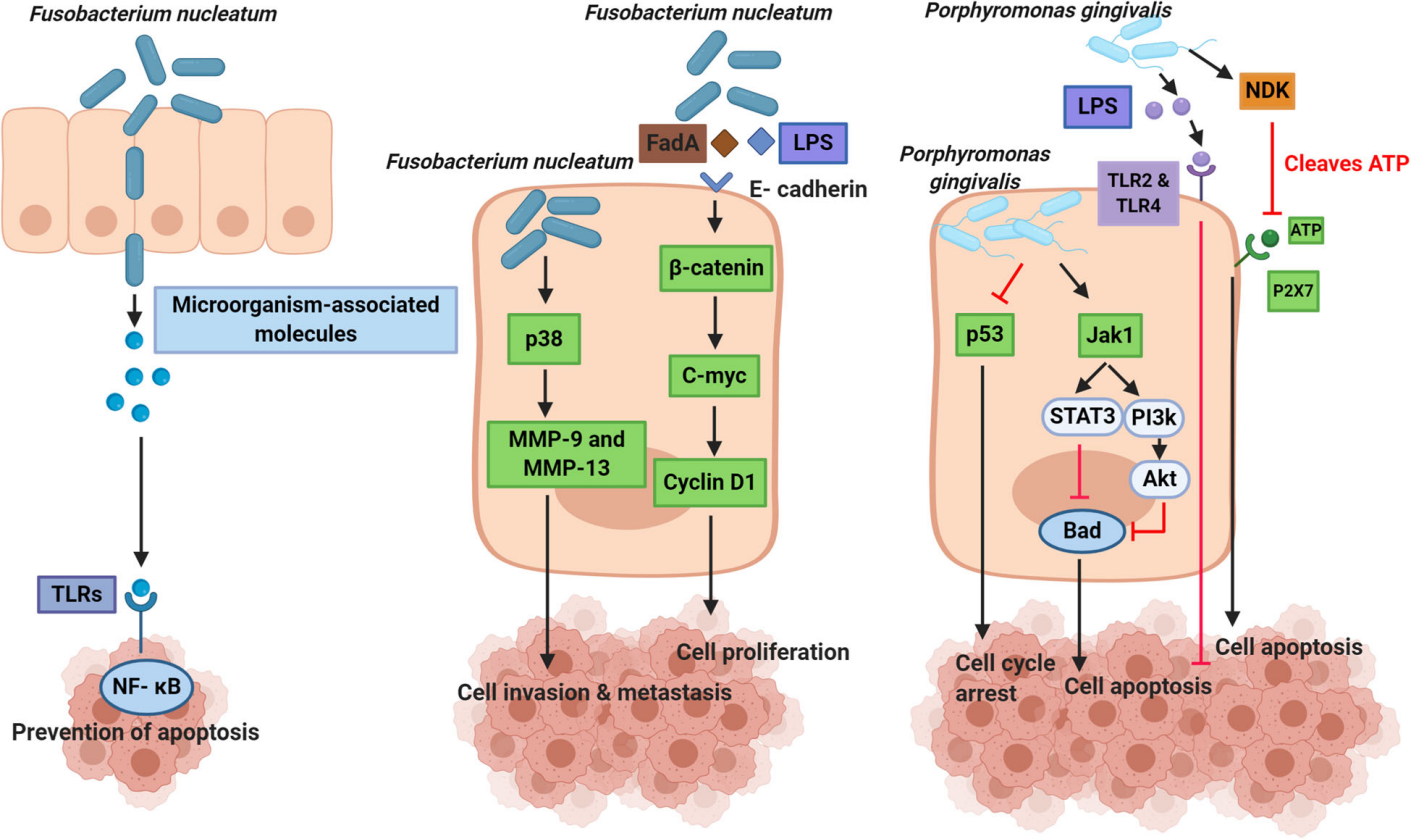
Oral bacteria can affect the inhibition of cell apoptosis. *F. nucleatum* modulates numerous anti-apoptotic pathways. As a consequence of TLR activation, bacteria stimulate NF-_k_B signaling. *F. nucleatum* activates p38, which results in the MMP-9 and MMP-13 secretion and leads to cancer cell invasion and metastasis. Also, *F. nucleatum* may induce β-catenin signaling by its LPS and FadA. Stimulating the β-catenin expression and increasing the expression of oncogenes C-myc and cyclin D1 lead to cell proliferation. *P. gingivalis* LPS may stimulate host response via TLRs (TLR2 and TLR4) and enhance the growth of tumor. Also, *P. gingivalis* induces anti-apoptotic Jak1/Akt/Stat3 signaling. This bacterium can secrete a NDK enzyme, which cleaves ATP and prevents the proapoptotic P2X7 receptor activation, thus modulating ATP/P2X7-signaling. It also causes cell cycle arrest by manipulating cyclin/CDK activity and reduced levels of p53. TLR: Toll-like receptor, NF- _k_B: nuclear factor kappa B, p38: Mitogen-activated protein kinase p38, MMPs: matrix metalloproteinases, LPS: lipopolisaccharide, FadA: fusobacterial adhesin/invasin, Jak1: Janus kinase 1, Akt: protein kinase B, Stat3: Signal transducer and activator of transcription 3, Bad: Bcl-2-associated death promoter, CDK: cyclin-dependent kinase, p53: Tumor protein p53, NDK: nucleoside diphosphate kinase, ATP: Adenosine triphosphate, P2X7: Purinergic receptor

Oral bacteria can affect the pathogenesis of cancers through influencing cytoskeletal rearrangements, inhibition of cellular apoptosis, cell proliferation, and activation of NF-_Κ_B [44]. *F. nucleatum* infection modulates numerous anti-apoptotic pathways. Toll-like receptor (TLR) activation causes bacteria stimulate NF-_k_B signaling [86]. FadA is a crucial pathogenic factor of *F. nucleatum* and changes methylation of the cyclin-dependent kinase inhibitor 2A (CDKN2A) promoter and infiltration of macrophage in cancer tissues [105]. *F. nucleatum* stimulates p38, which results in the MMP-13 and MMP-9 secretion and significantly affects cancer cell invasion and metastasis [106]. Also, *F. nucleatum* can induce signaling of β-catenin by its LPS. Enhancing the β-catenin expression and oncogenes C-myc and cyclin D1 is present in this process [90, 107]. The inflammatory cytokines activated by *F. nucleatum* LPS are IL-6, TNF-α, and IL-1β [44]. IL-6 may affect cancer development by influencing anti-apoptotic pathways [83]. *P. gingivalis* LPS may stimulate host response via TLRs, like TLR4 and TLR2 that may prevent apoptosis and enhance tumor proliferation; it therefore cooperates in the protection of tumor cells and the progression of cancer [84]. *P. gingivalis* functions anti-apoptotically through a lot of pathways’ modulation [108]. Intracellular *P. gingivalis* induces anti-apoptotic signaling of Jak1/Akt/Stat3 [109, 110]. This bacterium can discharge an anti-apoptotic enzyme nucleoside diphosphate kinase (NDK), breaking down ATP and inhibits the proapoptotic P2X7 receptor, thus modulating signaling of ATP/P2X7- [111]. It also accelerates progression via the cell cycle S-phase by manipulating cyclin/CDK activity; it decreases the p53 tumor suppressor level (Fig. 3) [112]. *P. gingivalis* results in significant pro-apoptotic Bad phosphorylation and inhibition, through enhancing the Bcl2 and Bax ratios [113].

#### Carcinogenic substances

Substances which are generated by oral bacteria with a carcinogenic effect consist of organic acids, volatile sulfur compounds (VSC), reactive nitrogen species (RNS) and reactive oxygen species (ROS), and hydrogen peroxide (H_2_O_2_). The *P. gingivalis* NDK secretion may modulate the ATP-induced cytosolic and mitochondrial ROS and the antioxidant glutathione response (AGR) generated via the P2X7/NADPH-oxidase interactome [114]. ROS can markedly activate inflammation/cancer-associated transcription factors [115]. In this process, some species in the oral cavity produce H2O2. The peroxigenic oral bacteria consist of *Streptococcus gordonii*, *S. oralis*, *Streptococcus sanguinis*, *S. mitis*, *Streptococcus oligofermentans* [116], *L. acidophilus*, *Lactobacillus fermentum*, *Lactobacillus minutus*, *Lactobacillus jensenii*, and *Bifidobacterium adolescentis* [117]. These findings emphasize the relationship between free radicals and chronic inflammation and their effect in developing cancer [118].

The microorganisms that metabolize alcohol to acetaldehyde significantly affects the cancer development. Oral bacteria (e.g., *Aggregatibacter actinomycetemcomitans*, *P. intermedia*, *P. gingivalis*, and *F. nucleatum)* generate VSCs including methyl mercaptan (CH_3_SH), dimethyl disulfide (CH3SSCH_3_), hydrogen sulfide (H_2_S), and dimethyl sulfide ((CH_3_)_2_S) [44]. VSCs are toxic to tissues and may develop chronic inflammation [119]. H_2_S is a common genotoxic agent and causes cumulative mutations or genomic instability [120]. H_2_S has dichotomous influences on many gastrointestinal processes like cancer, inflammation, and apoptosis [121].

Oral microbiota are able to metabolize alcohol (ethanol) to acetaldehyde, due to possessing the enzyme alcohol dehydrogenase (ADH), which is involved in carcinogenesis [88, 122]. It has been shown that several species of oral bacteria such as *S. mitis*, *S. gordonii*, *Streptococcus salivarius*, *S. sanguinis*, and *S. oralis* [123] possess ADH, which metabolizes alcohol to acetaldehyde [124] with a potential for cancer development [44]. Genus Neisseria can produce the large amounts of the ADH enzyme, which generates the carcinogen acetaldehyde, and along with *H. pylori* with high generation of this enzyme, may affect alcohol-related gastric carcinogenesis [122].

**Fig. 4:**
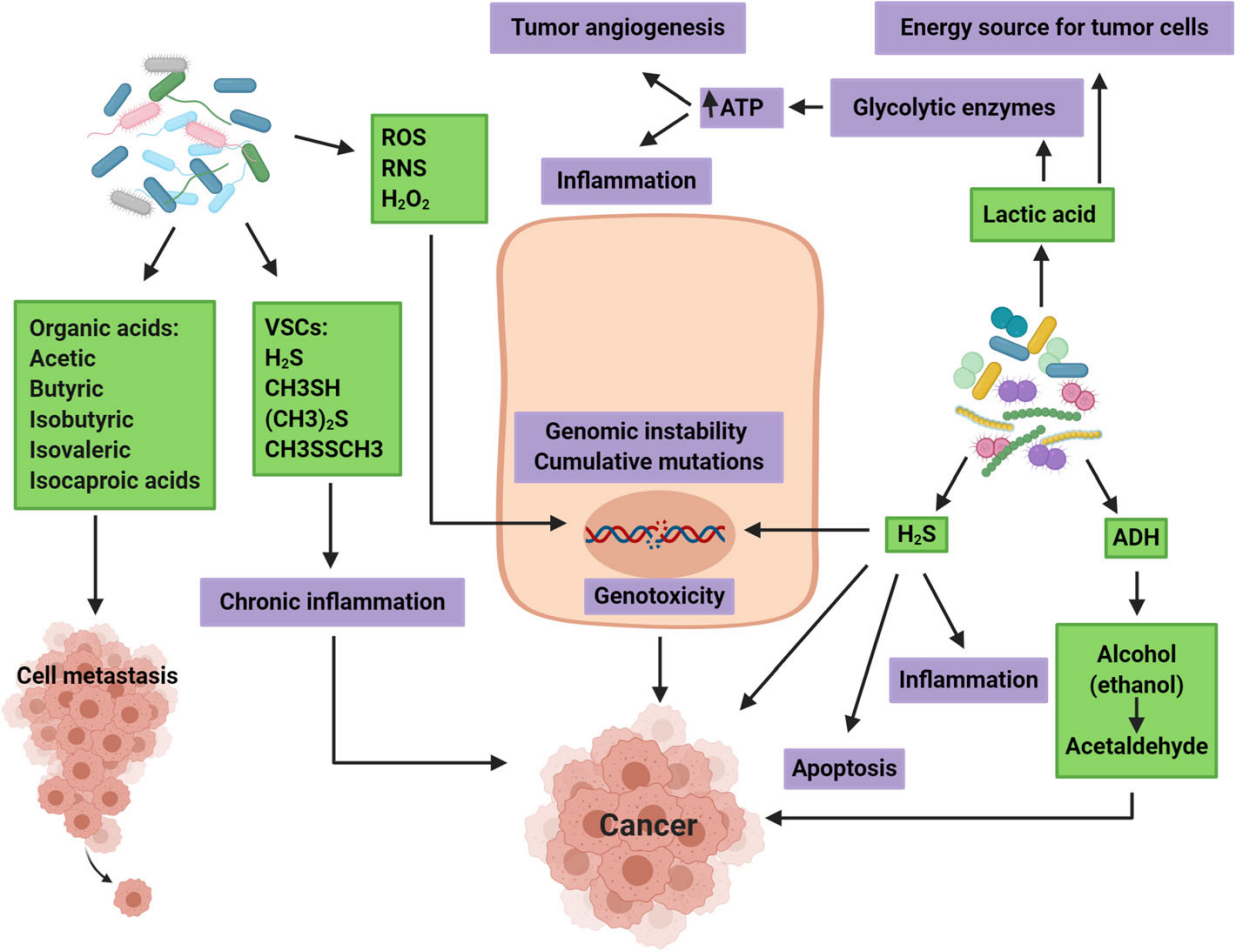
Oral bacteria produce some substances that play a role in chronic inflammation, genomic instability, tumor angiogenesis, and progression of gastric cancer. Some oral bacteria generate VSCs including CH_3_SH, H_2_S, CH3SSCH_3_, and (CH_3_)_2_S that may develop chronic inflammation. Oral bacteria are also involved in the production of ROS, RNS and H_2_O_2_, which may be involved in genotoxicity. Some species can generate organic acids (e.g., isobutyric, butyric, isocaproic, and isovaleric acids) that may contribute to cell metastasis. H_2_S may cause genomic instability, effects on inflammation, apoptosis, and many gastrointestinal processes like cancer. Other oral bacteria generate lactic acid, which is a source of energy for tumor cells and is involved in increasing ATP levels, which may exacerbate inflammation and angiogenesis. Some of them are able to metabolize alcohol to acetaldehyde by ADH enzyme, which is involved in carcinogenesis. VSCs: volatile sulfur compounds, CH_3_SH: including methyl mercaptan, H_2_S: hydrogen sulfide, CH3SSCH_3:_ dimethyl disulfide, and (CH_3_)_2_S: dimethyl sulfide, ROS: reactive oxygen species, RNS: reactive nitrogen species, H_2_O_2_: hydrogen peroxide, ATP: Adenosine triphosphate, ADH: alcohol dehydrogenase

Some species can generate acids more (e.g., aciduric *Peptostreptococcus stomatis* produces acetic, isocaproic, isobutyric, butyric, and isovaleric acids) [125]. Such acid production can affect the hypoxic and acidic microenvironment of the tumor, thus augmenting metastatic efficiency [126, 127]. Some oral bacteria of genera *Lactobacillus*, *Streptococcus*, *Bifidobacterium*, *Leuconostoc*, *Lactococcus*, and *Pediococcus* generate lactic acid [128]. These microorganisms are aciduric and acidogenic which may lower pH in the local environment by producing lactic acid [129]. *Lactobacillus* and *Lactococcus* species are known as probiotics and assumed good to the host. The production of lactic acid has immunomodulative, anti-inflammatory, and anti-cancer activities and contribute to *H. pylori* eradication [130–132]. Lactate also serves as energy source of the tumor, producing glycolytic enzymes to raise the supply of ATP. This metabolite may enhance inflammation and activate the angiogenesis of the tumor (Figs 4) [37–39, 133].

## Conclusion

Several factors, including tooth flossing [134], poor oral hygiene [135–138], the metabolism of oral microbes [78], and tooth loss [136–138] have been found to affect the risk of gastric precancerous lesions and gastric non-cardia carcinoma. Nevertheless, the causal correlation between oral microbiota and GC was not obvious. It is proposed that identifying specific oral microbiota proteins can help detect early GC. Therefore, cancer may be prevented by targeting and inhibiting oral carcinogenic microbial proteins or by eradicating certain microbiome species. More importantly, detecting the patterns of interaction between the oral cavity microbiota and *H. pylori* may render new clues for the diagnosis or screening of cancer. Integration of oral microbiota and *H. pylori* might manifest a potential method for the assessment of GC risk. Hence it needs to be specified the patterns of bacterial transmission from the oral cavity to the stomach and their interaction. Further evidence on the mechanisms underlying the oral microbiota communities and how they trigger GC may contribute to the identification of new prevention methods for GC. We may then modulate the oral microbiota by intervening with oral-gastric bacterial transmission or controlling certain bacteria in the oral cavity.

## Data Availability

Not applicable.

## Abbreviations

GC: Gastric cancer
*H. pylori*: *Helicobacter pylori*
CagA: Cytotoxin-associated gene A
GI: Gastrointestinal
*P. gingivalis*: *Porphyromonas gingivalis*
*P. intermedia*: *Prevotella intermedia*
16S rRNA: 16S ribosomal RNA
*S. mitis*: *Streptococcus mitis*
*S. oralis*: *Streptococcus oralis*
*S. anginosus*: *Streptococcus anginosus*
*S. marcescens*: *Serratia marcescens*
ANTTs: Adjacent non-tumor tissues
*L. lactis*: *Lactococcus lactis*
*L. brevis*: *Lactobacillus brevis*
LEfSe analysis: Linear discriminant analysis Effect Size
SG: Superficial gastritis
SCFAs: Short chain fatty acids
LPS: Lipopolysaccharide
*F. nucleatum*: *Fusobacterium nucleatum*
ADH: Alcohol dehydrogenase
*P. stomatis*: *Peptostreptococcus stomatis*
*P. micra*: *Parvimonas micra*
*D. pneumosintes*: Dialister pneumosintes
*S. exigua*: *Salix exigua*
MUC5B: Mucin 5B
MUC7: Mucin 5B
*F. periodontium*: *Fusobacterium periodontium*
SDSF: Streptococcus diffusible signal factor
AI-2: Autoinducer-2
*cag*PAI: *cag* pathogenicity island
*P. pallens*: *Prevotella pallens*
*P. histicola*: *Prevotella_histicola*
ERK1/2: Extracellular signal-regulated protein kinase 1/2
PCNA: Proliferating cell nuclear antigen
Egr-1: Early growth response protein 1
KEGG: Kyoto Encyclopedia of Genes and Genomes
IL-1β: Interleukin-1β
IL-6: Interleukin-6
IL-17: Interleukin-17
IL-23: Interleukin-23
TNF-α: Tumor necrosis factor alpha
MMPs: Matrix metalloproteinases
MMP-8: Matrix metalloproteinase-8
MMP-9: Matrix metalloproteinase-9
p21: A potent cyclin-dependent kinase inhibitor
p53: A tumor suppressor protein
TLRs: Toll-like receptors
FadA: Fusobacterium adhesin A
NF- _k_B: Nuclear factor kappa B
p38: Mitogen-activated protein kinase p38
NK: Natural killer
TIGIT: T cell immunoreceptor with Ig and ITIM domains
Fap2: Fusobacterium autotransporter protein 2
CDKN2A: Cyclin-dependent kinase inhibitor 2A
C-MYC: C-myelocytomatosis oncogene product
Jak1: Janus kinase 1
Akt: Protein kinase B, PKB
Stat3: Signal transducer and activator of transcription 3
NDK: Nucleoside diphosphate kinase
Bad: Bcl-2-associated death promoter
CDK: cyclin-dependent kinase
NDK: Nucleoside diphosphate kinase
ATP: Adenosine triphosphate
P2X7: Purinergic receptor
Bcl-2: B-cell lymphoma 2
BAX: BCL2-associated X protein
VSCs: Volatile sulfur compounds
RNS: Reactive nitrogen species
ROS: Reactive oxygen species
H_2_O_2_: Hydrogen peroxide
AGR: Antioxidant glutathione response
CH_3_SH: Including methyl mercaptan
H_2_S: Hydrogen sulfide
CH3SSCH_3_: Dimethyl disulfide
(CH_3_)_2_S: Dimethyl sulfide
*S. gordonii*: *Streptococcus gordonii*
